# Using Bayesian Belief Networks to Investigate Farmer Behavior and Policy Interventions for Improved Nitrogen Management

**DOI:** 10.1007/s00267-022-01635-6

**Published:** 2022-04-05

**Authors:** Felix Jäger, Jessica Rudnick, Mark Lubell, Martin Kraus, Birgit Müller

**Affiliations:** 1 Department of Ecological Modelling, Helmholtz Centre for Environmental Research – UFZ, Leipzig, Germany; 2 Department of Environmental Science and Policy, University of California Davis, Davis, CA USA

**Keywords:** Bayesian belief networks, Sustainable management practices, Farmer adoption, Agricultural decision-making, Policy analysis, Nitrate pollution

## Abstract

Increasing farmers’ adoption of sustainable nitrogen management practices is crucial for improving water quality. Yet, research to date provides ambiguous results about the most important farmer-level drivers of adoption, leaving high levels of uncertainty as to how to design policy interventions that are effective in motivating adoption. Among others, farmers’ engagement in outreach or educational events is considered a promising leverage point for policy measures. This paper applies a Bayesian belief network (BBN) approach to explore the importance of drivers thought to influence adoption, run policy experiments to test the efficacy of different engagement-related interventions on increasing adoption rates, and evaluate heterogeneity of the effect of the interventions across different practices and different types of farms. The underlying data comes from a survey carried out in 2018 among farmers in the Central Valley in California. The analyses identify farm characteristics and income consistently as the most important drivers of adoption across management practices. The effect of policy measures strongly differs according to the nitrogen management practice. Innovative farmers respond better to engagement-related policy measures than more traditional farmers. Farmers with small farms show more potential for increasing engagement through policy measures than farmers with larger farms. Bayesian belief networks, in contrast to linear analysis methods, always account for the complex structure of the farm system with interdependencies among the drivers and allow for explicit predictions in new situations and various kinds of heterogeneity analyses. A methodological development is made by introducing a new validation measure for BBNs used for prediction.

## Introduction

Nitrogen (N) pollution is one of the major causes of environmental degradation worldwide (Zhang et al. 2015). Agricultural fertilizer management contributes to N pollution in three major ways: leaching to groundwater, runoff to surface water, and volatilization in the atmosphere. To minimize nitrogen loss to the environment, farmers may implement certain ‘best management practices’ (BMPs) that aim to reduce nonpoint source pollution (Lubell and Fulton 2007). Despite research demonstrating BMP effectiveness, adoption rates for some practices remain low across farms (Ribaudo 2015; Rudnick et al. 2021; Wade et al. 2015). To increase efficiency of N management at a larger scale, it is crucial to understand the socio-economic factors and policy interventions that influence BMP adoption decisions.

However, despite decades of research, there is still much debate about which factors are the most important drivers of adoption (Prokopy et al. 2019; Ranjan et al. 2019). Results vary widely across contexts, specific management practices and analytical techniques. While there is some agreement that social network connections and farm size are important in most contexts, many other variables are not consistently positive or negative predictors for BMP adoption across different practices or farming contexts (Prokopy et al. 2008).

One promising leverage point for influencing adoption are outreach and extension activities where farmers learn about BMPs and gain awareness about nitrogen pollution (Emerson et al. 2012; Hillis et al. 2018). Enhancing outreach and extension is often more practically and politically feasible than other policy interventions such as fertilizer taxes or caps on applied N rates (Kanter et al. 2020). However, it is not clear how different forms of engagement will impact the adoption rate of different practices and their effectiveness may depend on farm characteristics. For example, outreach on BMPs may be more effective to farmers with large farms and more capital sources to support innovation adoption, or outreach may be more effective when tailored to specific farm types (Ma et al. 2012). Our analysis seeks to explore which outreach and engagement pathways ought to be considered viable options for policy interventions.

This paper applies a Bayesian belief network (BBN) modeling approach to analyze the drivers of N management practice adoption and the effects of policy interventions. BBNs are probabilistic models describing relationships of variables in systems. Using a network structure, BBNs allow for evaluation of which variables affect each other; these inter-variable dependencies are then modeled via conditional probability distributions. After entering an observation of one (or more) variables, the probabilities for the states of all other variables are updated using Bayes’ rule (Kjærulff and Madsen 2008). BBNs can be used for non-deterministic prediction in a system as well as investigation of influences of and interactions between system variables, while always taking account of uncertainty (Landuyt et al. 2013; McCann et al. 2006).

Key advantages to the BBN analytical approach in our study context include the ability for a network structure model to account for multiple interdependencies between the hypothesized predictor variables of BMP adoption. Most empirical studies of farmer adoption apply some type of linear model to assess the influence of different predictor variables on a farmers’ decision to adopt a practice. Typically, these models focus on the direct relation between predictor variables and the dependent variable. Therefore, they do not fully capture the interdependencies between the predictor factors that often drive adoption.

In addition, BBNs enable explicit predictions of adoption probability in new situations with limited observations or hypothetical external manipulations of predictor variables. This gives rise to the possibility of policy experiments (Celio and Grêt-Regamey 2016; Liehr et al. 2016), where specific variables within the model are manipulated to see the effect on otherwise observed data. Various kinds of heterogeneity analyses are also feasible, such as investigating the effect of policy measures across different types of farms. Thus, BBNs provide a useful tool for analyzing the relative importance of different adoption drivers, given the interdependencies between driver variables, and for predicting the adoption of N management strategies under different policy interventions. Further advantages of the method include the possibility of combining empirical data and expert knowledge (Landuyt et al. 2013; Uusitalo 2007), the ability to handle incomplete data cases (Aguilera et al. 2011) and the comprehensive graphical representation of causal relationships, which facilitates their use in participatory modeling (Haapasaari et al. 2013).

In environmental modeling, BBNs are rarely used and still show a lot of unexploited potential. As to our knowledge, the latest general review of BBNs in environmental modeling was presented by Aguilera et al. (2011). Initially, studies were mainly concerned with analyzing influences of environmental predictor variables on ecological response variables (Marcot et al. 2006), for example predicting coral bleaching (Done and Wooldridge 2004) or regeneration of an endangered Eucalypt species (Pollino et al. 2007a). However, more recently the method has found application in modeling environmental decision-making, such as the adoption of riparian management strategies (Ticehurst 2010), farm crop choice (Poppenborg and Koellner 2014), land use change (Aalders 2008; Celio and Grêt-Regamey 2016) or migration due to climate change (Drees and Liehr 2015).

This study disentangles the multiple, interdependent factors that drive farmers’ adoption of different BMPs and investigates the impact of policy measures on increasing adoption rates. The data comes from a survey on farmers’ behavior towards N management in the Central Valley of California. We consider characteristics of the farm (e.g. farm size and irrigation system type), and characteristics of the farmer, including both demographics (e.g. age and education level), and socio-behavioral variables such as participation in outreach/educational opportunities and information networks (henceforth, these activities are referred to as ‘engagement’). Furthermore, we examine the effect of three different policy measures related to strengthening farmers’ engagement, and evaluate their likely effect on farmer BMP adoption. A typology of farms, consistent with previous findings in the literature, is developed to compare the effect of the policy measures across farm types.

Our guiding research questions are:

- R1. What are the most important influence factors for the prediction of BMP adoption?
- R2. How do BMP adoption rates change under engagement-related policy interventions across different types of farms?

Our discussion reflects on our experiences with BBNs, including both the modeling capability potential, as well as its limitations in the context of analyzing influence factors of adoption of sustainable management practices. As the method is relatively new in this context (exceptions: Ticehurst 2010; Ticehurst et al. 2011), the discussion provides an important additional contribution to the literature.

## Study Area and Policy Context

This study is based on farmer survey data collected in the Central Valley of California. Agricultural use of nitrogen fertilizers has contributed to significant environmental pollution in California, particularly as a leading contaminant of groundwater with about 400,000 tons of nitrate leaching annually. Cropland and livestock are estimated to be responsible for 88% of the N leached to groundwater each year (Harter et al. 2012; Tomich 2016). A more sustainable approach to agricultural N management is crucial to improve the water quality.

California is a particularly interesting case study as it shows a great diversity in agricultural, ecological and economic factors related to farming. Its Mediterranean climate enables the growing of more than 400 commodity crops, both annual and perennial, across over 77,000 farming and ranching operations; however, dry summers mean a reliance on a highly engineered water system and cropland irrigation, making irrigation management practices a critical element of N management. The cultivated area amounts to 25 million acres of land, spread along a 500-mile longitudinal gradient (California Department of Food and Agriculture 2018). The Central Valley is California’s most productive agricultural region and one of the most productive in the world. From small family-owned farms to large international corporations, from more traditional to more innovative and technology-affine operations, a variety of different types of farms are represented. There are numerous potentially suitable N management practices; this study focuses on eleven of the most broadly applicable strategies (e.g. applicable to nearly all farm types), as identified by University of California Cooperative Extension agricultural advisors (Rudnick et al. 2021). A description of the eleven practices is given in Table 1.

**Table 1:** Description of the considered N management practices (Rudnick et al. 2021)

N management practice	Description
Soil testing	Test soil for residual nitrate at beginning of season and adjust fertilizer application rate as appropriate
Leaf testing	Test crop leaf for crop nutrient status to determine if plant is up-taking enough nutrients
Irrigation well N testing	Test irrigation water in wells for nitrate content and adjust fertilizer application rates as needed
Moisture probe	Test soil water content to determine depth of soil saturation and more precisely control irrigation to give crop just enough water, which still retaining fertilizer in root zone
Pressure bomb	Determine plant-water stress and adjust irrigation scheduling as appropriate, including when fertilizer is applied so that fertilizer stays in root zone
ET-based irrigation scheduling	Use evapotranspiration (ET) data to determine plant water losses, and calculate how much water needs to be replaced with irrigation. Appropriately place fertilizer in the irrigation set so that fertilizer stays in root zone
Split application	Divide fertilizer applications into smaller doses and apply in different applications at needed times in season
Fertigation	Apply fertilizer through irrigation sets, generally through drip irrigation system
Foliar application	Apply fertilizer directly to crop foliage (leaves); increases crop uptake and reduces fertilizer left in soil to be leached
Variable rate GPS	Vary rate of fertilizer application across a field using GPS technologies; allows targeting of specific regions/rows within field with appropriate rate given soil type and crop health
Cover crops	Plant cover crops to help hold moisture and nutrients in the soil; provides an organic source of nitrogen that breaks down more slowly over time

These practices are also some of the most important practices targeted by California’s Irrigated Lands Regulatory Program (ILRP), which was initiated in 2003 as a nonpoint source pollution abatement program, targeting agricultural impacts to water quality (Dowd et al. 2008). The ILRP has evolved and expanded in scope over time, and currently includes goals specific to N pollution including increasing farmers’ adoption of nitrogen management practices and increasing efficiency of nitrogen applications, with the objective of reducing nitrogen leaching to groundwater and runoff to surface waters. The ILRP applies a hybrid of regulatory and collaborative approaches to reduce the nitrogen loss on all irrigated farming operations. Farmers are obliged to report annually the N management practices they use and their N budget, a tool that tracks how much N was applied and removed in crop harvest. However, at present there is no obligation to implement certain practices nor a legal limit for nitrogen application. Instead, farmers are required to participate in local Water Quality Coalitions that serve as intermediators between individual farmers and the regulatory agency. These Coalitions assist the farmers in creating their annual N budget report, give advice on BMPs and organize education and outreach events. Moreover, they offer ‘Self-Certification’ courses which qualify the farmers to write their own N budget reports, which otherwise need to be verified by a certified crop consultant. Private crop consultants also do a lot of on-farm work to determine nutrient budgets and appropriate management practices.

The survey was carried out in 2018 in three Water Quality Coalitions: the Colusa Glenn Subwatershed Program (part of the Sacramento Valley Water Quality Coalition), the San Joaquin County and Delta Water Quality Coalition and the East San Joaquin Water Quality Coalition. A total of approximately 7,500 farming operations cultivating more than 900,000 acres of land is covered by these coalitions, which represent a longitudinal transect of about half the Central Valley. Much of the diversity of the state’s agricultural landscape is represented in this area.

In total there were 4994 surveys mailed across the three regions. With 967 responses, the response rate was on par with similar surveys. Farmer survey data was combined with anonymous ILRP reporting data where possible.

## Methods

### Construction of the Bayesian Belief Network

A BBN is a probabilistic model of a system of interrelated variables. A main use of BBNs is the non-deterministic prediction of a set of outcome variables given probability distributions of multiple predictor variables (Aguilera et al. 2011).

A BBN can be depicted as a network structure, consisting of a set of nodes, representing the system variables, and links between them, representing relationships between the nodes. Each node is assigned a finite set of mutually exclusive states (for example, ‘Farm Size’ is set to large (>200 acres), medium (50–200 acres) or small (<50 acres)). We considered three categories of variables; farm characteristics, farmer characteristics and engagement. Table 2 gives an overview of these variables, their meaning and the states used. All of them were queried in the survey.

**Table 2:** Influence variables considered in the study, meaning and states

Variable name	Description	States^a^
Farm characteristics		
Farm size	Size of the farm	Small (<50 acres), medium (50–200 acres), large (>200 acres)
Irrigation system	Does the farmer use a pressurized irrigation system on his largest field?	Yes/no
Crop type	Does the farmer grow perennial crops on his largest field?	Yes/no
Farmer characteristics		
Income	Annual gross farm-related income	Low (<50k$), medium (50k$–500k$), high (>500k$)
Education	Does the farmer have a college degree?	Yes/no
Years in farming	How many years is the farmer already engaged in farming?	Above/below median of 36 years
Engagement		
Self-certification	Is the farmer self-certified to write his own N budget report?	Yes/no
Number of information sources	Number of different information sources regarding N management used (aggregated from questions about single information sources including various public organizations, farm advisors, other growers, family members, field crew)	Above/below median of 4
Consultant	Does the farmer hire a consultant to help them complete their N management and farm evaluation plans?	Yes/no

^a^Continuous variables were discretized for use in the BBN; for ‘Income’ the states are based on the categories used in the United States Department of Agriculture Census of Agriculture, for ‘Farm Size’ the states are based on previous survey work at University of California Davis (UC Davis), for ‘Years in Farming’ and ‘Number of Information Sources’ we used the median as natural threshold

Farmers were also asked about their adoption of the eleven different N management practices of Table 1, measured as binary variables. We implemented the adoption of the considered practice (the dependent variable) as a target node in our network. That means, all the other variables point at the practice adoption node, either directly or indirectly through other intermediary nodes.

Beyond the network structure, in a BBN one needs to specify for each node a conditional probability table (CPT). Conditional probability describes the probability of an event given the outcome of another event. For example, the conditional probability that the income of a famer is “high” may be 60% under the condition that the farm size is “large”, or 30% when the farm size is small. The CPT provides the probability that the node is in a particular state, given that the nodes pointing on the chosen node are in a particular combination of states.

Once the BBN is set up in this way, from the CPTs it is possible to compute the probabilities of each node being in a particular state, without knowing the states of some or all of the other nodes. This allows for predicting likely behavior given only a limited set of observations. Additional observed data can be used to update the probability distributions of all the other nodes in the network. The underlying computations are based on Bayes’ rule, a mathematical law connecting different conditional probabilities.

An example of a CPT and the process of entering an observation in our BBN can be found in the Online Resource A and B.

The BBN was programmed into Netica 6.05 and all subsequent analyses were carried out with this software. The CPT of each node can either be defined explicitly by the user, follow fixed equations, or learned from data; unless otherwise stated we learned it from the California farmer survey data collected at University of California Davis. ‘Learning from data’ here means using an algorithm which gives out values for the conditional probabilities which fit to the observations in the data.

The choice of the variables as well as fixing the final network structure was done in an iterative process of constructing and evaluating networks (the evaluation measures used are explained in Section “Goodness of Fit and Model Validation” below). Empirical knowledge gained from prior studies of the IRLP was crucial in this procedure (Lubell and Fulton 2007; Rudnick et al. 2021).

**Fig. 1:**
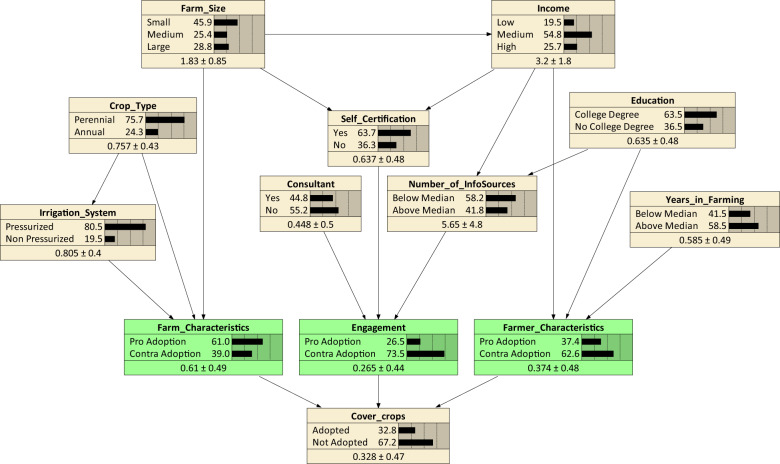
The Bayesian belief network structure with exemplarily ‘Cover crops’ as one of the eleven N management practices as target node

The final network contains the nine predictor variables from Table 2 and is depicted in Fig. 1. We evaluated the same network structure for each of the eleven management practices as target nodes.

The green nodes (‘Farm Characteristics’, ‘Farmer Characteristics’ and ‘Engagement’) are so-called intermediate nodes. They are artificial nodes forming a layer between the influence variables and the target node (a layer is a set of nodes with the same distance to the target node). We included them in order to reduce the number of links pointing on the target node; this is necessary as the CPT of a node grows exponentially with each additional link towards it (Marcot et al. 2006). If the CPT gets too large, the number of parameters that have to be learned from the data increases drastically. This leads to overfitting, which is expressed in poor goodness of fit scores and unreasonable predictions for cases that are not in the data.

In the Online Resource C, we explain how we gave the artificial nodes reasonable states.

All CPTs except for the target node’s CPT were learned from the data. To this end we used the Expectation-Maximization-Algorithm (Korb and Nicholson 2010) which can handle cases with missing data. The existence of this algorithm was crucial for our structure as it enabled us to use the artificial intermediate nodes with no data at all. Detailed explanations on setting the target node’s CPT can be found in the Online Resource C.

### Goodness of Fit and Model Validation

A Train and Test procedure was used to analyze the predictive power of the BBN and to evaluate its goodness of fit to the data set (Aguilera et al. 2011; Chen and Pollino 2012; Pollino et al. 2007b), thereby ensuring the model’s validation. In contrast to several scoring functions used in structure-learning algorithms (most prominent: the Bayesian information criterion) which assess the goodness of fit of a network structure to a data set (Abdulkareem et al. 2019; Beretta et al. 2018), using this procedure accounts for the numerical part of the BBN (the CPTs) and the measures are adapted to the BBN’s task of prediction. For each practice, the data was randomly split in two, a training data set containing 70% and a test data set containing 30% of the data. The former data set was used to learn the CPTs from, the latter to test the network’s capability to predict the target node. The most common measure for prediction accuracy is the Error Rate: For each case in the test data set, all data values except for the target node value (here practice adoption) were entered in the network and the most likely state of the target node under these assumptions was calculated (using the usual inference in a BBN). If this predicted state was not the true state from the data, it was counted as an error. The share of errors in all the cases in the test data set is the Error Rate.

To avoid statistical outliers the Error Rate was averaged over five random partitions for each target node. This is often referred to as Cross-Validation (Aguilera et al. 2011).

However, the Error Rate alone has only limited meaning when it comes to the question of how adequately the influence dynamics are modeled in the BBN, especially if networks with different target nodes are compared. The reason is that the Error Rate highly depends on the actual adoption rate of the respective target variable. We illustrate this with an example: Let A and B be two N management strategies with adoption rates P(A) = 40% and P(B) = 5%. If we just use two ‘trivial’ networks containing only the target node (i.e. base the prediction only on the adoption rate), both predict ‘no adoption’ in every test case as this is the most likely state. Though the network structure is equal, the first network then has an Error Rate of 40%, while the second predicts wrong in only 5% of the cases. This shows that the Error Rate is highly correlative with the adoption rate of the target node. Now, if a more complex network has an Error Rate of 10%, this can be considered very good if the target node is A, but if the target node is B, it is worse than predicting always the same, based only on adoption rate and independent of all other influence variables. In the latter case, we cannot assume the influence dynamics of the system are modeled in the right way.

To overcome these issues and provide a better validation of the model, we introduce the following new measure which quantifies the improvement of the prediction of the network compared with prediction based only on the adoption rate. We term it ‘Prediction Improvement Value (PIV)’:

**PIV**: The difference between the Error Rate from prediction based only on adoption rate and the Error Rate of the network (=min(AdoptionRate, 1 – AdoptionRate) – ErrorRate), with high PIV indicating a highly accurate network

The PIV is still biased by the adoption rate of the target node: Networks with target node adoption rate near 50% generally have a higher PIV than networks with very low or very high target node adoption rates: For the latter, prediction is likely to be always “no” or always “yes”, regardless of the predictor variables. Hence, they can hardly distinguish adopters from non-adopters and considering them doesn’t improve prediction much. In any case the sign of the PIV can serve as an important indicator for goodness of fit of a BBN designed for prediction: A negative PIV means that the predictions of the model are worse than predicting independently of all influence variables, based only on adoption rate, hence the influence patterns aren’t modeled adequately. This can happen for example due to too little data.

In our study we used three levels of reliance according to the PIV: (i) not meaningful (negative PIV), (ii) reliable (PIV between 0% and 10%) and (iii) especially reliable (PIV > 10%).

At the beginning of our analysis we excluded the practices for which the model was not meaningful (PIV < 0%) from the following investigations. For the other practices we consider the model as validated, conceptually by expert knowledge during the construction process and numerically by the satisfactorily high PIVs.

### Analysis Tools and Policy Experiments

First, we analyzed the importance of different influence factors for practice adoption (research question R1) by means of a sensitivity analysis. For each target node we let the CPTs be learned from the whole data set and recorded the sensitivities of the target node to the predictor variables, using the measure Mutual Information. Mutual Information is a commonly used measure of sensitivity of a variable to findings in another variable in BBNs (Chen and Pollino 2012; Kleemann et al. 2017a; Ticehurst 2010), further explanations can be found in the Online Resource D. Average sensitivities over all target nodes were calculated.

Second, we conducted experiments for potential policy measures regarding the engagement variables while also examining the effect of the interventions on adoption rates across different types of farms (research question R2). We restricted ourselves to the practices whose adoption was predicted especially reliably by the model (PIV > 10%). Here we will first explain how we implemented the interventions and then how we implemented the groupings of farms.

We looked at three different policy measures regarding the three engagement variables ‘Consultant’, ‘Self-Certification’ and ‘Number of Information Sources’ and implemented these measures, each in two different versions:

- **Full intervention**: We set the state on ‘yes’ (for ‘Consultant’ or ‘Self-Certification’) or on ‘above median’ (for ‘Number of Information Sources’). Therefore, after the intervention all farmers make use of the respective engagement channel.
- **Normalized intervention**: We added only 10% (in absolute percentage points) to the ‘yes’/‘above median’ state for the respective engagement variable. That represents 10% of the farmers changing from the ‘unengaged’ to the ‘engaged’ state.

The analysis of both types of interventions leads to different, but complementary insights: The full interventions indicate the whole potential that lies in manipulating the respective variable to the fullest degree. However, they lack a certain comparability as they don’t take into account which share of farmers already is in the ‘engaged’ state. If for example, only 20% of the farmers hire a consultant, the ‘Consultant’ full intervention makes 80% of the farmers change their behavior. This is likely to have much stronger effect (and also would take much more effort) than it would be the case if already 90% of the farmers hired a consultant. The normalized interventions are used to eliminate this effect.

The absolute change in percentage points of the predicted adoption probability caused by the respective intervention was measured to evaluate its effect.

Finally, we assessed the effect of these interventions across different types of farms. The BBN allowed for a simple implementation of this kind of heterogeneity analysis. Farms were grouped by the combinations of their three defining farm characteristics: size, crop type and irrigation system. Putting together small and medium farms this leads to eight groups we abbreviate with 3 letters, the first for ‘Farm Size’ (S = small/medium, L = large), the second for ‘Crop Type’ (P = perennial, A = annual), the third for ‘Irrigation System’ (P = pressurized, N = non-pressurized). An LPP farm for example is a **L**arge farm with **P**erennial crops and a **P**ressurized irrigation system. Table 3 shows the number of farms in each group. LPP, SPP and SAN are the biggest groups, so we decided to focus on them and added LAN to be able to better compare -PP with -AN and L– with S– farms. This typology is consistent with previous literature which identifies farms growing perennial crops with a pressurized irrigation system as innovative in contrast to farms with annual crops and a non-pressurized irrigation system (Rudnick et al. 2021). For each of these four groups the above six different policy measures (full and normalized interventions for ‘Consultant’, ‘Self-Certification’ and ‘Number of Information Sources) were investigated by additionally entering the respective farm characteristics as observations in the network. This way we could quantify how much the practice adoption probability increases due to a political intervention, given that the farm is of some particular type.

**Table 3:** Number of farms for each farm type

Farm class	Farm size	Crop type	Irrigation system	Number of farms
LPP	Large	Perennial	Pressurized	161
LPN	Large	Perennial	Non-pressurized	5
LAP	Large	Annual	Pressurized	32
LAN	Large	Annual	Non-pressurized	46
SPP	Small/Medium	Perennial	Pressurized	406
SPN	Small/Medium	Perennial	Non-pressurized	89
SAP	Small/Medium	Annual	Pressurized	76
SAN	Small/Medium	Annual	Non-pressurized	99

## Results and Discussion

### Methodological Pre-analysis on Goodness of Fit (Prediction Improvement Value analysis)

**Fig. 2:**
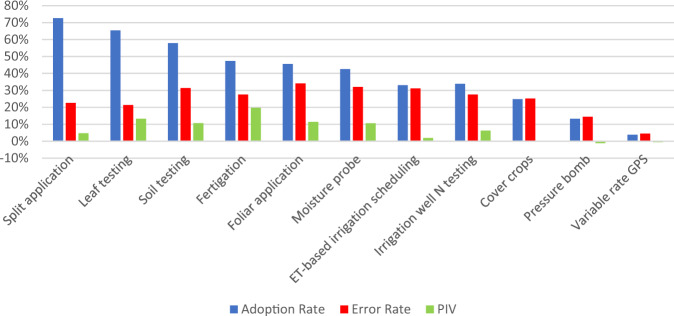
Adoption rates, Error Rates and prediction improvement value (PIV) per practice

Figure 2 shows the adoption rates, Error Rates and PIVs of the BBNs for each of our eleven practices of interest. As discussed in the methods section, the Error Rate highly depends on the adoption rate of the practice, where it is highest for practices with adoption rate close to 50%. Correspondingly, the PIV is also highest for those practices as the improvement potential is larger. Regarding the direction of the PIV, we observe that most target nodes have positive PIV, meaning that prediction with the BBN is better than predicting independently of the influence variables, based only on adoption rate. Nevertheless, there are three practices with negative PIV, which can happen, for example, due to too little data, especially when adoption rate is so low that the predictor variables cannot distinguish adopters from non-adopters. We excluded ‘Cover crops’, ‘Pressure bomb’ and ‘Variable rate GPS’ from the following analyses as they have PIV < 0%; but we assume our model to have explanatory power for the remaining practices. Values indicating special reliability (PIV > 10%) were found for ‘Leaf testing’, ‘Soil testing’, ‘Fertigation’, ‘Foliar application’ and ‘Moisture probe’, so these were selected for the policy experiments in Section “Effect of engagement-related policy experiments across different farm types”.

### Importance of Influence Factors for BMP Adoption

**Fig. 3:**
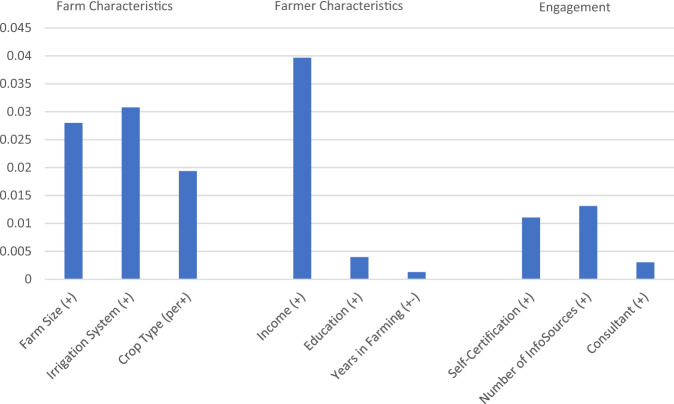
Mean sensitivity of the target node to the predictor variables over the considered practices (measure: Mutual Information). Direction of influence on adoption is indicated in brackets behind variable names: + positive influence, - negative influence, +- not consistent; for ‘Crop Type’ the state’per’=‘perennial’ increases adoption probability

First, we examined the general influence of the predictor variables on practice adoption (research question R1). We investigated the mean sensitivities of the target node to the predictor variables, which are depicted in Fig. 3, together with the direction of influence, i.e. whether a variable increases or decreases adoption probability. A table with the sensitivity data for each individual practice can be found in the Online Resource E.

The three categories of variables defined in the methods section will guide the closer analysis.

Farm characteristics are consistently the most important of the three categories.’Irrigation System’ and’Farm Size’ demonstrate similarly high sensitivity values and rank second and third, respectively, as influence factors; ‘Crop Type‘ ranks fourth in influence.

This confirms results found in Rudnick et al. (2021) that highlight the importance of structural farm variables in determining the feasibility of BMP adoption. It also corroborates past farmer adoption literature that explains the relationship between structural farm variables and adoption: Farm size correlates with access to capital and technical expertise and provides an economies of scale advantage in adoption (Caswell et al. 2001; Daberkow and McBride 2003). Pressurized irrigation systems show more innovation and access to technical capital, additionally some of the considered BMPs were co-evolved with pressurized irrigation systems (Hanson et al. 2009; Taylor and Zilberman 2017). Perennial cropping systems indicate long-term thinking as well as greater access to financial resources (Blank 2001; Ghadim et al. 2005; Marra et al. 2003).

The farmer characteristics show varying importance: ‘Income’ is the most reliable indicator for adoption of all considered variables. ‘Education’ and ‘Years in Farming’ are less consistent predictors of adoption, and rank behind the structural farm characteristics variables.

Income not only indicates access to financial capital to overcome the upfront cost of adoption, but also strongly correlates with farm size (Prokopy et al. 2019). The finding about ‘Education’ and ‘Years in Farming’ is consistent with the broad literature reviews of farmer adoption literature in the USA, where these variables don’t always have consistent impact on predicting adoption (Baumgart-Getz et al. 2012; Prokopy et al. 2008; Prokopy et al. 2019).

The engagement variables rank in the lower half of all predictor variables, regarding their sensitivities. ‘Number of Information Sources’ still shows a similar sensitivity value as ‘Crop Type’, followed by ‘Self-Certification’. ‘Consultant’ is one of the least important influence factors of all variables considered.

Access to (and quality of) information has been found a particularly important variable across many studies (Baumgart-Getz et al. 2012; Prokopy et al. 2019). The rather low influence of ‘Self-Certification’ and ‘Consultant’ can partially be explained together with the finding that these variables show great heterogeneity across management practices. We hypothesize that not all the considered practices receive attention in the ‘Self-Certification’ courses or in the consulting of the N management advisors, respectively. Thus, these variables show high sensitivity values only for some of the practices.

In a second step, we compared the sensitivities of adoption to the predictor variables for different management practices. The sensitivities for each practice are presented in the Online Resource E. Most of the findings above are consistent over all the practices; for example, ‘Farm Size’ and ‘Income’ are almost always among the top three most decisive variables, whereas ‘Years in Farming’ and ‘Education’ never play a notable role. Nevertheless, for some practices significant deviations from the general influence pattern could be observed, especially when it comes to the role of the engagement variables. For example, for ‘Foliar application’, the sensitivities are quite low in general, but ‘Number of Information Sources’ has a surprisingly high sensitivity value. For the ‘Soil testing’ practice, ‘Crop Type’ and ‘Irrigation System’ are not as important, but ‘Self-Certification’ has a comparatively high influence on adoption. This heterogeneity is due to technical idiosyncrasies of the individual practices and the (practical) knowledge spread in the different forms of engagement possibilities. For instance, the finding for ‘Soil testing’ may be explained by the fact that testing the soil for residual nitrate is independent of the crop type and irrigation system while it requires expertise to interpret the data accordingly. This specialized knowledge may be gained mainly from the progressive Self-Certification courses.

We can conclude that in our case the basic influence dynamics towards BMP adoption can be described as follows: ‘Income’ and the structural farm characteristics are the most important variables and mainly suffice for prediction. Engagement plays a complementary role. However, we focus the policy experiments on the engagement variables as they are easiest to influence and do still have a notable influence on adoption.

### Effect of Engagement-related Policy Experiments Across Different Farm Types

Next, we present the results of the policy experiments described in Section “Analysis tools and policy experiments”. First, we investigated the general effect of increasing farmers’ engagement on adoption of different practices using the previously mentioned six different policy interventions; second, we compared the effect across the different types of farms. The five considered practices are the ones with particularly convincing modeling performance of the BBN (see Section “Methodological pre-analysis on goodness of fit (Prediction Improvement Value analysis)”).

**Fig. 4:**
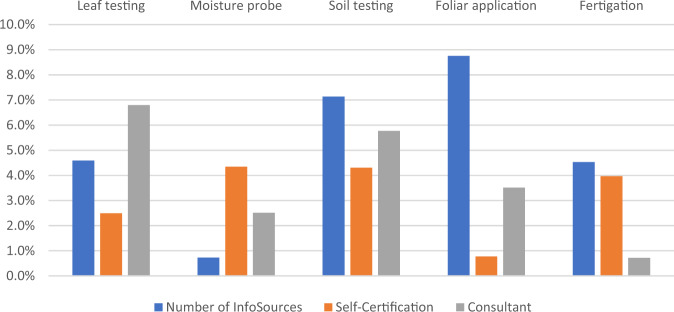
Absolute change of adoption rate in percentage points for each full intervention for each considered practice

Figure 4 shows the absolute change of the adoption rate for each practice for the full intervention (where all farmers are set to the ‘engaged’ state of the resp. variable).

The influence of the interventions is heterogeneous across different types of practices. Increasing the number of information sources strongly increases the adoption of ‘Foliar Application’ and ‘Soil testing’ but has almost no effect on the adoption of ‘Moisture probe’. The ‘Self-Certification’ full intervention seems to be fruitful for ‘Soil testing’ and ‘Fertigation’; on the other hand, it doesn’t increase the adoption of ‘Foliar Application’. Hiring an N management consultant leads to much higher adoption of ‘Leaf testing’ and ‘Soil testing’ but has almost no effect on the adoption of ‘Fertigation’. The picture for the normalized interventions (where only 10% of farmers change to the ‘engaged’ state) looks similar, but with smaller absolute impacts (see Online Resource F). This mirrors our earlier finding that the single engagement variables can vary strongly in their influence on adoption across practices. The reasons lie in the idiosyncrasies of the different forms of engagement. For instance, the last finding about the ‘Consultant’ intervention may be explained by the fact that consultants are often the ones to take samples for ‘Leaf testing’ and ‘Soil testing’ and make their product recommendations based on the tests. ‘Fertigation’ instead doesn’t require a consultant, and rather is implemented directly by the farm or irrigation manager; thus, adding a consultant may do little to influence adoption of this practice.

This suggests that policy interventions should be tailored to the specific challenges and features of different practices.

**Fig. 5:**
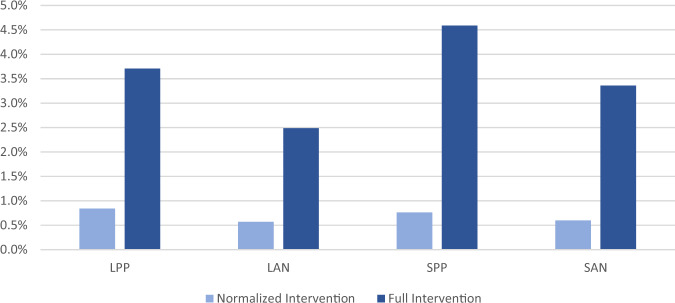
Absolute change of adoption rate in percentage points for the full and the normalized interventions (all farmers are set to the ‘engaged’ state vs. only 10% change to the ‘engaged’ state) per farm type (averaged over all practices and engagement channels)

Finally, we look at the effect of policy interventions across the different types of farms, averaged over all considered practices and engagement channels, but for both the full and the normalized interventions (Fig. 5; the full individual data for each practice and each intervention can be found in the Online Resource F). For this we use the typology of farms developed in the methods section classifying farms along two dimensions: large farms versus small farms (L- vs. S- farms) and farms with perennial crops and a pressurized irrigation system versus farms with annual crops and non-pressurized irrigation (-PP vs. -AN farms), resulting in the four different farm types LPP, LAN, SPP, and SAN.

Comparing -PP with -AN farms, the effect on adoption rates of all interventions is always greater in the -PP farms than the -AN farms. According to the authors’ experience, farms that have a pressurized irrigation system and grow perennial crops are more technologically and economically sophisticated than -AN farms, potentially indicating higher all-around levels of innovation. Our overall finding on the effect of interventions suggests that innovative and well-resourced farmers may be more responsive to increased engagement activities than less-resourced farmers in terms of BMP adoption rates. This may be due to the fact that there is more outreach tailored to -PP farms, with practices adapted to best suit -PP farms and field days often featuring trials on -PP farms, so these better respond to an increase of farmers’ engagement. Another explanation may be that by virtue of their overall attitude towards and capacity for innovation, innovative farmers are more capable of learning and incorporating new knowledge. In this way, increased engagement for innovative farms leads to heightened learning on the BMPs under consideration, thereby increasing adoption.

If one compares large and small farms under the policy interventions, the picture is less clear. The effect of the normalized interventions on BMP adoption is not consistently higher for either. However, farmers with large farms already show a greater engagement (79% of the farmers with large farms are self-certified vs. 47% of the farmers with small farms, ‘Number of Information Sources’: 54% vs. 36%, ‘Consultant’: 38% vs. 42%). Thus, putting all farmers in the ‘engaged’ state of the respective engagement variable is less of a change from the observed data for large farms than for small farms. Therefore, regarding the full interventions, the farmers with small farms respond better to the measures.

Altogether our results suggest that it is essential to fit the policy intervention to the targeted nitrogen management strategy and to the farm type that is targeted for adoption. Strengthening the engagement possibilities in their current form has more effect on farmers with -PP farms than on farmers with -AN farms, indicating that an adaptation of these possibilities may be needed to better suit the -AN farms. Such a general statement cannot be made about different sizes of farms, but farmers with small farms show more unexploited potential in increasing their engagement and thus adoption.

### Discussion of the Method

BBNs were a helpful tool for investigating the influence factors on BMP adoption. However, they require a cautious approach for various reasons. We discuss the advantages and limitations of using BBNs in the given research context.

#### Advantages

BBNs always take the whole causal structure of the system into account. In contrast to more conventional statistical methods such as regression models, interdependencies between predictor variables are modeled explicitly. Thus, BBNs align with farm systems thinking, i.e. thinking of the multitude of agronomic, economic, ecological, and socio-behavioral factors influencing decisions on a farming operation.

BBNs enabled us to quantify the effect of external policy interventions on practice adoption in a natural, reasonable way. They are particularly useful for prediction of explicit probability distributions of the target variable in various scenarios (Celio and Grêt-Regamey 2016; Drees and Liehr 2015). This focus on explicit prediction of adoption probability has several other advantages: For example, it allows for applying a fully calibrated BBN to predict adoption in new contexts where fewer data are available. Moreover, BBNs could be used to model the farmers’ decision-making part in related agent-based models (ABMs) (Pope and Gimblett 2015; Sun and Müller 2013). Such an incorporation in an ABM would allow an investigation of the dynamics over time and, by coupling with biophysical models, to explicitly consider the social-ecological feedbacks in the system, such as the evolvement of N content in soil or groundwater over time, depending on specific policy interventions.

At the same time as entering the policy interventions in the model, we could also specify characteristics of the farm under consideration. Hence, the method allowed us to compare the effect of policy measures across different farm types and therefore to carry out predictive analyses that are limited under traditional linear statistical analysis. Moreover, we could assess the importance of influence factors across different practices by just changing the target node. Therefore, we found BBNs a flexible tool for different kinds of heterogeneity analyses.

While conducting our study, we discovered another analysis method BBNs offer which is the comparison of the predictive power of different network structures (Done and Wooldridge 2004). Though in the end we didn’t include them in our study, we initially investigated multiple network structures and consider this a promising approach for further research. By analyzing which network structures better explain the observed data, new knowledge about the role of and interactions between predictor variables may be derived.

Finally, as often, when survey data is used, we had to cope with a lot of incomplete data cases. For the artificial intermediate nodes there was no data at all. It turned out an important advantage of BBNs that there are reliable algorithms for learning the CPTs which are able to handle cases with missing data (Uusitalo 2007). Nevertheless, it should be kept in mind that extrapolating data can have a large impact on the simulated outcomes of the model and replaces a complete data set only at the prize of higher uncertainty.

#### Limitations

The most difficult step in the construction of our BBN was fixing the network structure. The structure influences the results in a non-negligible way; however, often multiple structures are found equally reasonable. Compared with a conventional statistical analysis, this adds a dimension of uncertainty to the results. In our case, we relied heavily upon the use of theory and expert knowledge for fixing the structure. This agrees with the experience made in other studies dealing with BBNs in an environmental context (Kleemann et al. 2017a; Uusitalo 2007). Though we tested an algorithm to learn the structure of the BBN from the data, the resulting structures were not reasonable in any way (e.g. ‘Farm Size’ pointing on ‘Education’) and did not show better goodness of fit values either. Other sources recommend to use structure learning only in combination with expert knowledge and when a high amount of data is available (Alameddine et al. 2011; Kleemann et al. 2017b; Sun and Müller 2013). Another technical issue we encountered while constructing the network structure is the need for avoiding too many links pointing onto the same node due to the risk of overfitting (see 3.1). Artificial intermediary nodes partially solved this problem in our study (Drees and Liehr 2015).

Care has to be taken not only while building the network structure but also when comparing different networks in the analysis. We already pointed out that the Error Rate is a good indicator for the predictive power, but not that appropriate for evaluating the goodness of fit, if networks with different target nodes are compared. Instead we introduced a new measure, the PIV, which we consider more meaningful when it comes to the question which networks represent the influence dynamics well. The idea of comparing the Error Rate of the model with the prediction accuracy of a comparative method was already established in Drees and Liehr 2015; yet, without explicitly explaining the methodological issue behind it. The PIV gives a natural way to overcome the bias of the Error Rate and is ready to be used in further research due to the detailed description provided in this paper. However, the PIV is still biased by the adoption rate of the target node (see Section “Goodness of fit and model validation”).

Additionally, the sensitivities of the target node to the variables in different network structures should not be compared without further ado. They strongly depend on the number of layers as well as the total number of variables in the network (Ticehurst 2010).

Further disadvantages of using BBNs in the given context include the need to discretize continuous variables such as farm size and income which reduces information granularity (Uusitalo 2007). It is also quite difficult to model feedback loops and add temporal and spatial dimension (Landuyt et al. 2013). This becomes important if one wants to closer investigate the long-term biophysical impacts of political interventions on the environment and could be better dealt with in an ABM (Pope and Gimblett 2015; Sun and Müller 2013).

## Conclusion

Our analyses showed that income and farm characteristics consistently are the most important drivers for BMP adoption in our case study. Access to financial and technical capital appear to be limiting factors driving adoption decision-making, and thus show to be more important drivers of adoption than access to outreach and engagement or other socio-behavioral traits of the farmer. This is consistent with Rudnick et al. 2021 results based on the same data, but using linear regression methods.

Our findings suggest that BBNs might be useful to understand and predict BMP adoption behavior in other contexts where less data exists. For example, our results suggest that by knowing farm characteristics and income alone, data that is frequently collected in agricultural censuses, we may be able to fairly reliably estimate the likelihood of adoption of many BMPs. The BBNs’ strength of prediction under uncertainty underlies this benefit. Nevertheless, the ambiguity of results in previous research on the topic indicates that local idiosyncrasies have to be considered when a detailed picture of the situation is needed.

Our findings indicate that there are important differences in the factors that influence adoption on different practices, especially when it comes to the importance of different forms of farmers’ engagement. These are likely related to technical properties of the practices itself and the design of N management programs or education. Accordingly, our results from the policy experiments show that the effect of engagement-related policy measures strongly depends on the considered practice. This indicates that policy measures should especially be targeted to the specific practices. To gain understanding on specific targeting of practice adoption via policy instruments, the development of a sophisticated grouping of nitrogen management practices according to the crucial factors of influence may be an interesting pathway for further research.

Beyond offering strong analytical tools for modeling observed data, BBNs also let us explore and model the effect of possible policy interventions, including how they may differentially impact different farm types. This is a new contribution to previous research. Engagement-related policy interventions consistently have a higher effect on BMP adoption on farms which may already be classified as “innovative” – more technologically advanced with higher value crop types. This finding suggests policy measures should consider adapting the outreach and extension opportunities to better target less-innovative farmers, who traditionally have been underserved by extension. This could result in better addressing the barriers these farmers face during adoption and ensuring that all practices are suitable for all types of farms.

Regarding the farm size, there is no consistently different effect of the considered interventions on adoption on small or large farms, but as farmers with small farms currently show lower engagement, there is more potential of increasing their engagement and thus adoption than for farmers with large farms where the engagement level is already very high and likely more difficult to increase. However, one should keep in mind that we are only looking on change of adoption rates and not at the area where the BMP is implemented.

A more holistic evaluation of the environmental effects of policy measures targeting N management practices is needed. This could be achieved by a coupled spatial socio-environmental model; for instance, by linking an ABM, dynamically simulating policy measures and behavior change, with a biophysical model, estimating the effect on environmental pollution. We consider this an important and promising task for future research.

## Supplementary Information

Supplementary Material ESM
