# Supplementary material for: Using Bayesian Belief Networks to Investigate Farmer Behavior and Policy Interventions for Improved Nitrogen Management
Source: Environ Manage. 2022 Apr 5;69(6):1153–66. doi: 10.1007/s00267-022-01635-6 (PMC9079016; doi:10.1007/s00267-022-01635-6)
Supplement: Supplementary file 1 — Supplementary Material ESM [file 267_2022_1635_MOESM1_ESM.pdf]

Online Resource A-F

Supplementary information to the paper

Using Bayesian belief networks to investigate farmer behavior and policy interventions for improved nitrogen management

Journal: Environmental Management

Authors: Jäger, Felix[1*]; Rudnick, Jessica[2]; Lubell, Mark[2]; Kraus, Martin[1]; Müller, Birgit[1]

[1]Helmholtz Centre for Environmental Research – UFZ, Department of Ecological Modelling, Leipzig, Germany
[2] University of California Davis, Department of Environmental Science and Policy, Davis, USA
*Corresponding author, e-mail address: felix.jaeger@ufz.de

A. Example of a conditional probability table (CPT)

Table A1 shows an example CPT of the node 'Self-Certification' in the complete network. 'Self-Certification' has the two states 'Yes' and 'No'. The nodes with links pointing at 'Self-Certification' are 'Income' and 'Farm Size' with the states 'low'/'medium'/'high' and 'small'/'medium'/'large', respectively. For each of the nine possible combination of these states there is a row in the CPT which specifies the corresponding conditional probabilities for 'Self-Certification'. For instance, if the farmer in the model has low income and medium farm size, he is self-certified with a probability of around 73%.

Table A1 Example CPT of the node 'Self-Certification' in the complete network

Income	Farm_Size	Yes	No
Low	Small	50.368	49.632
Low	Medium	73.267	26.733
Low	Large	99.999	1.20e-3
Medium	Small	63.216	36.784
Medium	Medium	60.078	39.922
Medium	Large	75.88	24.12
High	Small	9.76e-4	99.999
High	Medium	64.39	35.61
High	Large	74.357	25.643

B. Example of the entering of an observation in a BBN

Figure B1 and Figure B2 show the complete network first without and then with an observation entered. In this case we examine the network when the state of 'Income' is known to be 'high'. Using the CPTs, Netica is able to compute new probabilities for the states of the other nodes given this information. For example, the adoption probability of 'Cover crops' rises from 32.8% to 39.9%. Also, the probability distributions of nodes pointing at 'Income' change: When the farmer's income is observed to be high, it is much more likely that the farm size is large (28.8% to 78.6%).

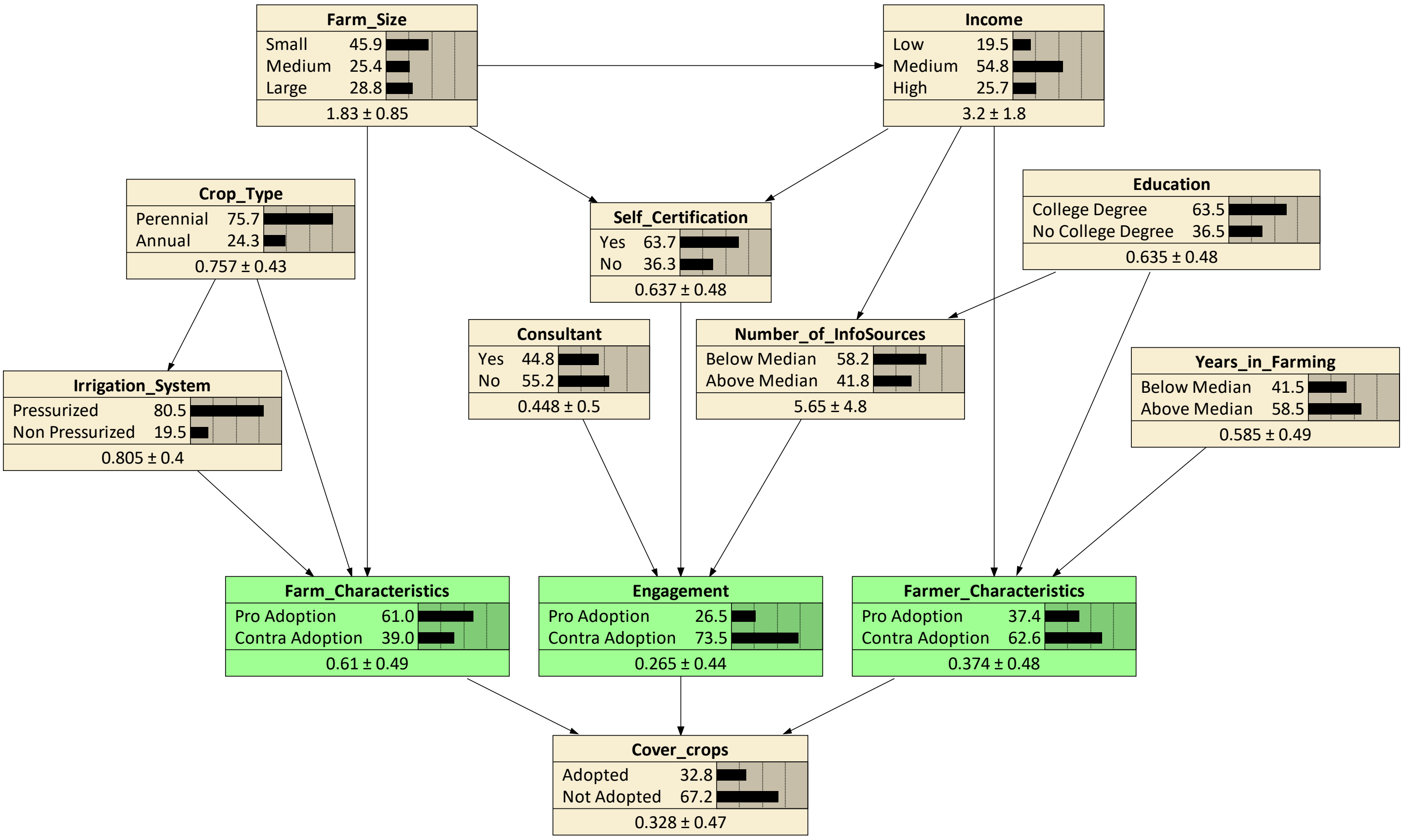

Figure B1 The Bayesian belief network

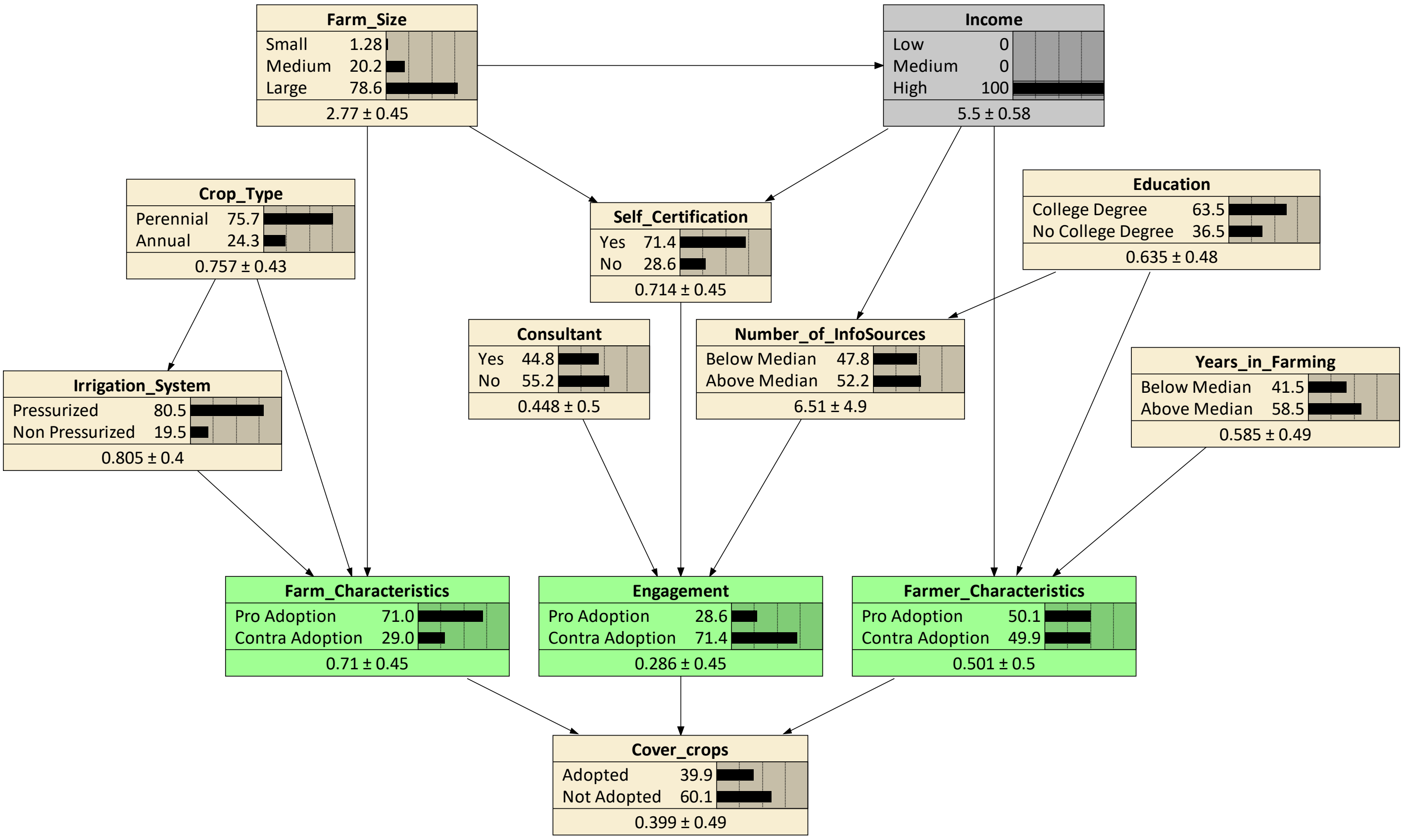

Figure B2 The Bayesian belief network with an entered finding for ‘Income’

C. Conditional probability table of the target node

For the artificial intermediate nodes, we decided to use two states for each, 'pro adoption' and 'contra adoption'. If now all CPTs were learned from the data, there would be no reason for the calculated probabilities to reflect the meaning of these designations, as the corresponding variables don't appear in the data. To ensure the meaningfulness of the states, we set the CPT of the target node manually in such a way that the designations make sense (Table C1).

Table C1 Conditional probability table of the target node in the complete network

Farm_Characteristics	Engagement	Farmer_Characteristics	Adopted	Not Adopted
Pro Adoption	Pro Adoption	Pro Adoption	100	0
Pro Adoption	Pro Adoption	Contra Adoption	50	50
Pro Adoption	Contra Adoption	Pro Adoption	50	50
Pro Adoption	Contra Adoption	Contra Adoption	25	75
Contra Adoption	Pro Adoption	Pro Adoption	50	50
Contra Adoption	Pro Adoption	Contra Adoption	25	75
Contra Adoption	Contra Adoption	Pro Adoption	25	75
Contra Adoption	Contra Adoption	Contra Adoption	0	100

The more intermediate nodes are in the state 'pro adoption', the higher is the adoption probability for the target node. The chosen probabilities are kept symmetric in the different intermediate nodes in order to not additionally bias the results. Alternative variants for setting the target node's CPT were checked and found not to change the results significantly.

The CPTs of the intermediate nodes can be learned from the data, although there isn't any data for the corresponding variables. The Expectation-Maximization-algorithm (the chosen learning algorithm) takes into account the manually set CPT as well as the data for the target node and calculates CPTs for the intermediate nodes in such a way that the influence of the other variables on the target node is modelled adequately.

D. Explanation of the sensitivity measure Mutual Information

The sensitivity measure Mutual Information used in this paper has its origin in a branch of mathematics called Information theory. It can be described as follows: Given two random variables X and Y, Mutual information measures the information one gets about X by observing Y. It is defined as $H(X) - H(X|Y)$ where $H(X)$ denotes the entropy of X and $H(X|Y)$ the entropy of X conditioned on Y. The entropy of X can be seen as a measure of uncertainty of the outcome of a realization of X or, equivalently, the expected amount of information gained by a realization of X. It is maximal for uniformly distributed random variables and zero for constant random variables. $H(X|Y)$ measures the uncertainty of the outcome of a realization of X if the outcome of Y is already known. The difference of these two then can be seen as a measure of how much of the uncertainty of X vanishes if Y is observed, or how much information about X one already gains by observing Y. Explicit definitions of entropy and conditional entropy can be found in any introduction to Information Theory.

E. All sensitivities for all practices

Table E1 Sensitivities of the target node to the predictor variables per practice (measure: Mutual Information) and rank of the variables according to sensitivity per practice (behind the slash). Sensitivity values >0,02 are green, sensitivities <0,005 red. CT='Crop Type', IS='Irrigation System', FS='Farm Size', I='Income', YiF='Years in Farming', E='Education', C='Consultant', SC='Self-Certification', NIF='Number of Information Sources'

	Leaf testing	Moisture probe	Split application	Irrigation well N testing	Soil testing	Variable rate GPS	Foliar application	Fertigation	Cover crops	ET-based irrigation scheduling	Pressure bomb	**Mean**
CT	0,038 /2	0,016 /5	0,028 /2	0,010 /5	0,009 /6	0,000 /7	0,020 /3	0,010 /6	0,009 /4	0,023 /4	0,010 /4	**0,019 /4**
IS	0,031 /4	0,031 /3	0,044 /1	0,022 /3	0,011 /5	0,000 /5	0,019 /4	0,054 /1	0,011 /3	0,033 /2	0,013 /3	**0,031 /2**
FS	0,034 /3	0,037 /2	0,019 /4	0,023 /2	0,042 /2	0,001 /3	0,014 /5	0,030 /3	0,013 /2	0,025 /3	0,034 /1	**0,028 /3**
I	0,058 /1	0,051 /1	0,026 /3	0,023 /1	0,047 /1	0,001 /4	0,028 /1	0,050 /2	0,014 /1	0,035 /1	0,024 /2	**0,040 /1**
YiF	0,000 /9	0,003 /6	0,003 /7	0,001 /9	0,002 /8	0,000 /7	0,002 /9	0,000 /8	0,000 /8	0,000 /9	0,000 /8	**0,001 /9**
E	0,001 /8	0,000 /9	0,000 /9	0,020 /4	0,000 /9	0,000 /7	0,003 /6	0,002 /7	0,004 /6	0,006 /7	0,000 /9	**0,004 /7**
C	0,010 /7	0,001 /8	0,000 /8	0,002 /8	0,007 /7	0,002 /2	0,002 /7	0,000 /9	0,000 /9	0,001 /8	0,003 /7	**0,003 /8**
SC	0,011 /6	0,019 /4	0,006 /6	0,006 /7	0,020 /3	0,000 /6	0,002 /8	0,016 /4	0,002 /7	0,009 /6	0,008 /5	**0,011 /6**
NIF	0,013 /5	0,003 /7	0,014 /5	0,008 /6	0,019 /4	0,005 /1	0,023 /2	0,011 /5	0,004 /5	0,015 /5	0,003 /6	**0,013 /5**

F. Full results of policy experiments

Table F1 Absolute change of adoption rate in percentage points for the full interventions for each considered practice per farm type; C, S, N denote the interventions related to the engagement variables 'Consultant', 'Self-Certification' and 'Number of Information Sources' respectively; farm types are coded as follows: first letter: farm size, L=large, S=small/medium; second letter: crop type, P=perennial, A=annual; third letter: irrigation system, P=pressurized, N=non-pressurized

Full	Leaf testing			Moisture probe			Soil testing			Foliar application			Fertigation		
	C	S	N	C	S	N	C	S	N	C	S	N	C	S	N
All	6,8%	2,5%	4,6%	2,5%	4,3%	0,7%	5,8%	4,3%	7,1%	3,5%	0,8%	8,8%	0,7%	4,0%	4,5%
LPP	7,0%	1,6%	3,8%	2,9%	3,7%	0,9%	4,9%	3,2%	7,5%	3,5%	0,3%	8,3%	-0,8%	3,4%	5,5%
LAN	3,6%	0,8%	2,0%	1,7%	2,1%	0,6%	4,5%	3,0%	7,0%	2,1%	0,2%	5,1%	-0,5%	1,9%	3,2%
SPP	7,6%	3,1%	5,4%	2,6%	5,0%	0,7%	6,5%	5,0%	7,5%	3,9%	1,0%	9,8%	1,3%	4,6%	4,7%
SAN	4,9%	2,1%	3,6%	2,0%	4,0%	0,6%	4,8%	3,7%	5,5%	2,9%	0,8%	7,2%	1,1%	3,6%	3,7%

Table F2 Absolute change of adoption rate in percentage points for the normalized interventions for each considered practice per farm type; C, S, N denote the interventions related to the engagement variables 'Consultant', 'Self-Certification' and 'Number of Information Sources' respectively; farm types are coded as follows: first letter: farm size, L=large, S=small/medium; second letter: crop type, P=perennial, A=annual; third letter: irrigation system, P=pressurized, N=non-pressurized

Norm.	Leaf testing			Moisture probe			Soil testing			Foliar application			Fertigation		
	C	S	N	C	S	N	C	S	N	C	S	N	C	S	N
All	1,1%	0,6%	0,7%	0,4%	0,9%	0,1%	1,0%	0,9%	1,1%	0,6%	0,2%	1,4%	0,1%	0,9%	0,6%
LPP	1,2%	0,7%	0,7%	0,5%	1,5%	0,2%	0,8%	1,3%	1,4%	0,6%	0,1%	1,5%	-0,1%	1,4%	1,0%
LAN	0,6%	0,3%	0,4%	0,3%	0,9%	0,1%	0,8%	1,2%	1,3%	0,4%	0,1%	0,9%	-0,1%	0,8%	0,6%
SPP	1,3%	0,6%	0,8%	0,5%	1,0%	0,1%	1,1%	1,0%	1,1%	0,7%	0,2%	1,4%	0,2%	0,9%	0,6%
SAN	0,8%	0,5%	0,6%	0,3%	0,8%	0,1%	0,8%	0,8%	0,9%	0,5%	0,2%	1,2%	0,2%	0,8%	0,6%
